# Dental Developmental Stages and Decayed, Missing, and Restored Teeth in Neurofibromatosis Type 1-affected Children and Adolescents

**DOI:** 10.4317/jced.61363

**Published:** 2024-03-01

**Authors:** Reinhard E. Friedrich, Moritz Schön

**Affiliations:** 1 Department of Oral and Craniomaxillofacial Surgery, Eppendorf University Hospital, University of Hamburg, Hamburg, Germany

## Abstract

**Background:**

Neurofibromatosis type 1 (NF1) is an autosomal dominant inherited tumor predisposition disease with a highly variable phenotype. The influence of the characteristic NF1 tumors (neurofibromas) on dentition has not yet been examined in detail. The aim of the study was to assess the dentition of NF1 children and adolescents, considering the symmetry of tooth development.

**Material and Methods:**

The panoramic radiographs of 59 patients with a confirmed NF1 diagnosis were compared with 59 age-and-sex-matched controls. The stages of tooth development on the sides of the jaw, added to a score, were assessed. In addition, the number of filled or decayed teeth, and the number of retained or missing teeth were assessed.

**Results:**

The tooth development of both study groups is symmetrical for almost all parameters and in the same developmental stage according to the sum score of the tooth development stages. Discrete developmental delays of teeth, in particular in the oral area of facial plexiform neurofibroma (PNF) are noticeable. NF1 patients’ teeth showed less decay and more restorations than that of the control group. The facial PNF (FPNF) does not impair emergence of deciduous teeth.

**Conclusions:**

Development of dentition of NF1 patients does not differ from the general population. However, FPNF with oral tumor components often prevent mesial movement of permanent molars and premolars, so these teeth do not develop contact (spacing), hardly emerge or may stay retained in bone. Oral PNF may have a low-retarding effect on some tooth root development (e.g., wisdom teeth). This effect is negligible when comparing the affected and unaffected sides of the jaw and is probably non-specific.

** Key words:**Neurofibromatosis type 1, plexiform neurofibroma, dentition, mixed dentition, symmetry, oral health, tooth development.

## Introduction

Neurofibromatosis type 1 (NF1) is an autosomal dominant inherited disorder characterized by a plethora of findings and symptoms (1). Prevalence of NF1 is about 1:3000 individuals living at birth (1). NF1 diagnosis is based on a panel of recently updated (clinical) findings (2) (Table 1). Tumors arising from peripheral nerve sheath cells, termed neurofibroma, are the hallmark of the disease (3). Gene causing NF1 is located on chromosome 17. The best-known function of the gene product (neurofibromin) is a tumor suppressor activity effective in the regulation of pathways of human homologue to rat sarcoma (RAS) gene (4). However, NF1 phenotype implies more than a tumor predisposition syndrome. Neurofibromin is involved in cellular and tissue differentiation (5). Therefore, the alterations of many tissues and organs in NF1 justify the alternative description of NF1 being a histogenesis control gene (6) (Table 1).

**Table 1:**
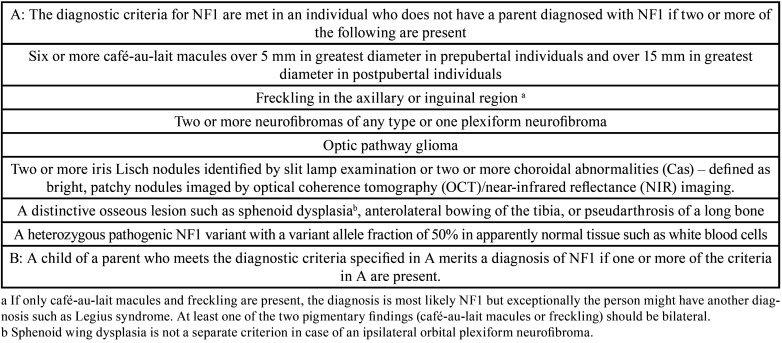
Revised diagnostic criteria of neurofibromatosis type 1 (NF1) (2).

Studies on dental development and oral health in NF1 provide conflicting results. Some authors identified acceleration of deciduous dentition in affected children (7), whereas another population-based study could not substantiate such conclusions, both in deciduous and permanent teeth (8). In addition, estimates of dental health status differ in several studies on NF1-affected individuals (9-12).

The aim of this study was to measure developmental stages of teeth and dental health in young NF1 patients using well-established examination techniques in dentistry. The dental findings of the NF1 patient group were examined considering the influence of the type of peripheral nerve sheath tumor (PNST) in the region of interest.

## Material and Methods

Radiographs. Fifty-nine orthopantomogram (OPG) images of 59 patients (female: 24, male: 35) with clinically confirmed NF1 (2) were evaluated (NF1 group = NFG) (Table 1). These 59 radiographs were compared with 59 OPGs of a randomly selected reference group (RG, n=59) from the patient files of the Radiology Department, University Dental Clinic (Head: C. Scheifele, DMD). Both age and sex were matched in patient group (NFG) and RG. Only OPGs were evaluated for RG, which were prepared for clarification of a potential orthodontic treatment. Patients with known syndrome, history of maxillofacial surgery, or known facial trauma were excluded from the selection process. Exclusion of OPGs from evaluation for technical reasons was due to projection-related divergences such as shadowing, overlapping, distortion or a tooth axis deviation perpendicular to the projection plane. This means that of the original 72 NFG patients, the X-rays of 59 met the examination standards. In both groups (RG, NFG) each individual tooth (position) was considered.

The age of the patients ranged from 3 to 18 years (ys). The mean age of both the entire groups was 10.32 years (females: 12 ys; males: 9.17 ys).

Emphasis was placed on examining the calcification of the teeth, root growth, and morphology of the apex. In addition, the number of teeth/missing teeth, decayed and filled teeth, presence of tooth germs, and impacted teeth were considered. A total of 16 parameters were evaluated for each tooth individually. Permanent and deciduous teeth were considered separately for evaluation. Analysis of symmetrical tooth development stages and health status of the RG’s deciduous and permanent dentition has been described in detail elsewhere (13).

Dental examination parameters. Criteria assessing dental health (caries, missing teeth, fillings, retained) and development (stages) were applied as detailed elsewhere (14-16). The finding “retained” was also applied in wisdom teeth.

**Figure 1:**
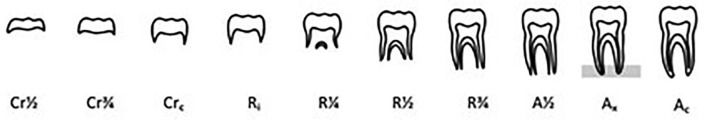
Modified growth stages classification of this study (13,17,18).

For each tooth, the developmental status was assessed and registered in a numerical code. For the purposes of this study, a modified classification of dental developmental stages proposed by Gleiser and Hunt was applied (13,17,18) (Fig. 1).

Scoring of tooth development. The individual developmental stages were assessed and registered in binary form: 0 = does not apply; 1= applies. For further calculations, following the individual assessment, the sum score of developmental stage was determined from the values Cr½ to Ac per jaw sides. Only one development stage in the individual case could apply. The higher the value of the score, the more advanced the tooth was developed (13).

Evaluation. Ten of 16 classification criteria addressed dental growth stage (Fig. 1) and were summed to the total score. If a tooth was classified ‘missing’, ‘germinated’ or ‘partially resorbed’, the tooth was not considered in the sum score. Since the influence of NF1 and specifically that of a FPNF on tooth development was to be investigated, the right-to-left side comparison was performed considering location of FPNF (tumor side vs. non-affected side in FPNF patients). FPNF is always unilateral in this study. The intra-individual comparison of the values investigated the impact of tumor side on dental findings.

Nomenclature of teeth. Féderation Dentaire International (FDI) tooth classification and diagram was used for tooth numbering and graphical representation of a patient’s finding (19).

Type of peripheral nerve sheath tumor (PNST). A further evaluation considered type of PNST in NFG. Cutaneous neurofibroma are the hallmark of NF1 (20). These tumors arise in the skin and are limited in extent to a maximum of a few centimeters. Cutaneous neurofibroma do not develop invasive growth, for example in muscle or bone. In NF1 patients, the tumors very often occur disseminated in the integument including the face and oral mucosa (disseminated (cutaneous) neurofibromas = DNF). The tumors are usually not noticeable until after puberty. An influence on dentition is considered unlikely. However, plexiform neurofibroma (PNF) can develop to large size. PNF arising in the face may cause severe orofacial disFiguring and dental problems. PNF are considered congenital tumors and precancerous. Therefore, the NFG was further differentiated into those patients who had developed facial PNF (FPNF) and those who had no FPNF. All patients could have DNF, but only FPNF patients were counted in FPNF group. FPNF often have an impact on bone development, in that the tumors are associated with conspicuous deformities of the jaws (21). FPNF are in almost all cases unilateral findings that may extend to the midline of the affected half of the face. In this study, at least the second and/or third trigeminal branches had to be affected by PNF. The influence of FPNF on tooth development is investigated both against patients with DNF and intra-individually comparing FPNF-affected vs. FPNF-unaffected body side. Differentiation into FPNF or DNF group was based on clinical findings (including numerous surgical and histological reports) and evaluation of sectional imaging, predominantly magnetic resonance imaging of the head and neck region (22). The influence of the facial tumor type on radiological findings of NF1 patients’ jaws has been very striking in previous studies and should therefore also be evaluated for the examination of dentition and dental health of this age group (22).

Statistics. Data were digitally recorded and evaluated (ExcelTM, (Microsoft Corp., Redmond, WA, USA); SPSS™ (IBM, Armonk, NY, USA)). Cross tabulations were created based on the data collection and significance was determined using the Chi-Square test. Fisher’s exact test was applied in values lower than 5. A difference *p*<0.05 was recorded as significant. The t-test was used for the statistical evaluation of growth stages. To test the hypothesis that significant differences exist between the study groups, the independent sample test was used to calculate mean differences. Mean comparisons were also used to determine which group had higher or lower scores on each scale. the significance level was set at < 5%. First, the reference group (RG) was examined for symmetry of tooth development, tooth change, and oral health care indices (13). Second, the overall groups (RG, NFG) were compared with respect to all 16 parameters regardless of sex. The comparison was jaw-side specific. Subsequently, NFG was compared with RG in all 16 listed parameters considering sex and jaw side (body side). Finally, the NFG was further evaluated with the facial tumor type defining the subgroup. Dental findings were calculated considering PNS tumor type (FPNF vs. DNF) and side of FPNF (tumor side vs. non-affected side).

## Results

A total of 6,136 teeth/tooth positions were considered (3,068 teeth per study group, considering potential 32 permanent teeth and 20 deciduous teeth). Number of missing teeth was 889 (RG) and 865 (NFG; *p*=0.613). A total of 4,382 teeth were evaluated.

The first part of the study focuses on tooth development in the RG and on the effects of gender. Another aspect is the examination of the symmetry of the stages of tooth development in the comparison of the jaw halves. Then the dental findings of the patient group are subjected to the same analysis. The dental findings of NFG were further analyzed considering evidence of FPNF (DNF vs. FPNF). Finally, the evaluation of FPNF subgroup’s tooth development was performed considering the PNF-affected jaw side.

The presentation focuses on key findings.

1. Reference group (RG)

The tooth development stages of deciduous and permanent dentition of RG indicated symmetrical development. Likewise, the radiological parameters of dental health were symmetrically distributed (*p*>0.05, n.s.). The sexual dimorphism of human dentition with significantly earlier tooth change in female subjects was confirmed. The results are presented elsewhere in detail (13) (Table 2,  Table 2 cont.).

**Table 2:**
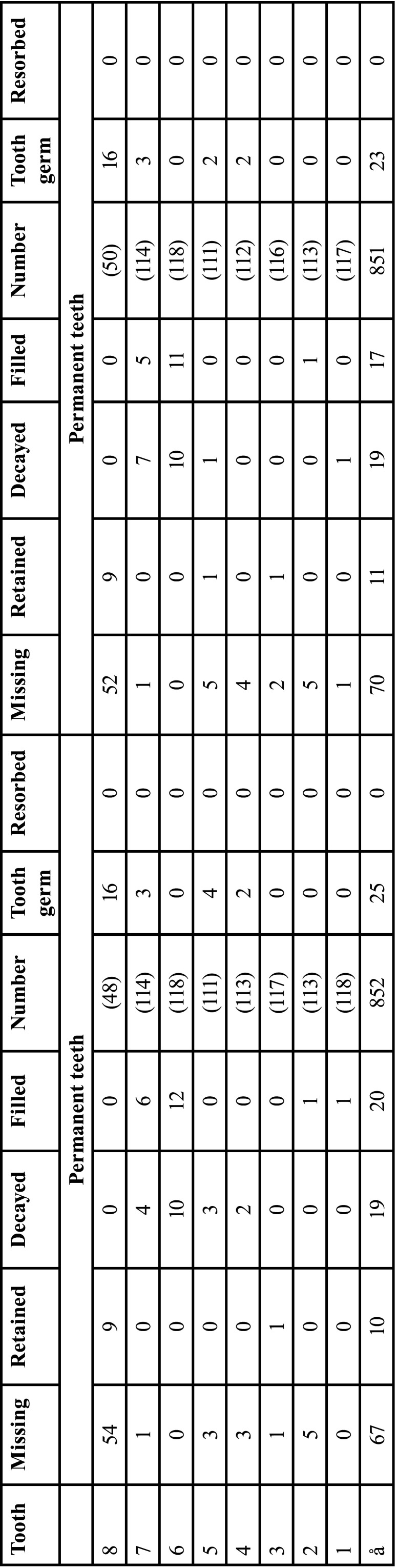
Findings of deciduous and permanent teeth of the reference group. Left and right sides of the jaws are compared (right side = 1. and 4. Quadrant (Q), left = 2. and 3. Q). Identification of teeth: Number 1-8 address permanent teeth, M1-M5 deciduous teeth (in ascending order from anterior to distal, starting with the central incisor (1, M1), applicable for all tables). Neither dental findings nor developmental stages show statistically significant differences related to body side.

**Table 2 cont.**
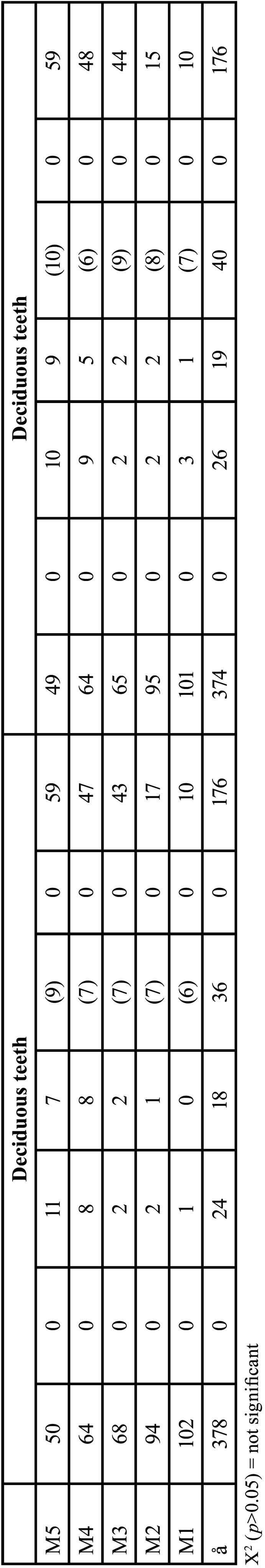
Findings of deciduous and permanent teeth of the reference group. Left and right sides of the jaws are compared (right side = 1. and 4. Quadrant (Q), left = 2. and 3. Q). Identification of teeth: Number 1-8 address permanent teeth, M1-M5 deciduous teeth (in ascending order from anterior to distal, starting with the central incisor (1, M1), applicable for all tables). Neither dental findings nor developmental stages show statistically significant differences related to body side.

2. RG vs. NFG

2.1. Total group comparison independent of sex

NFG and RG were compared regarding 16 dental evaluation criteria. The evaluation was side-specific and did not consider the sex. Deciduous and permanent teeth were compared separately. The term “jaw side(s)” implies findings of both jaws of one body side (Table 3,  Table 3 cont.).

**Table 3:**
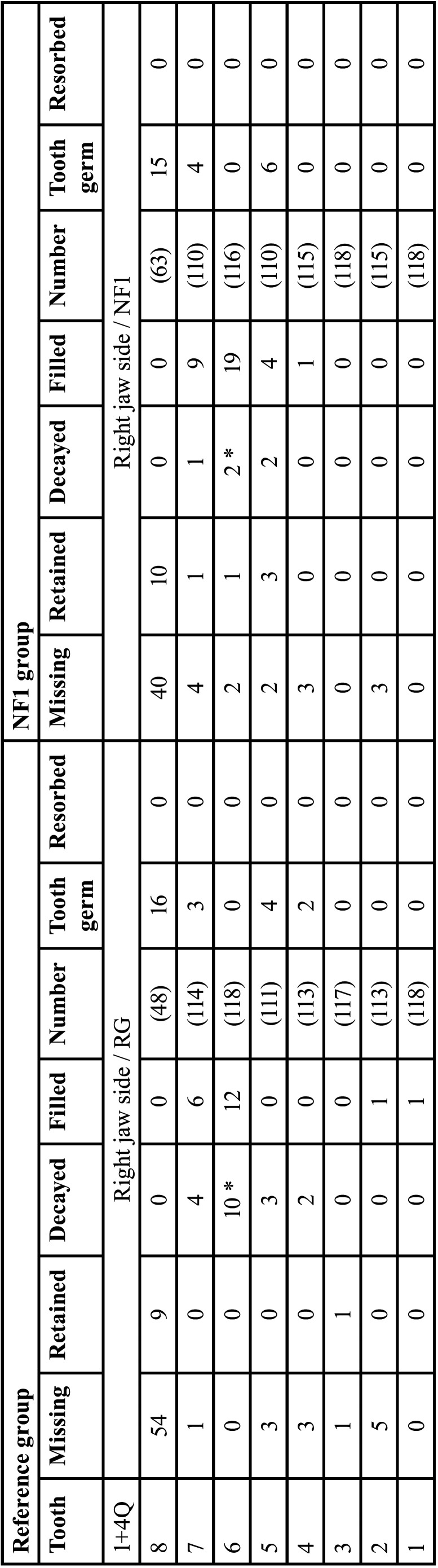
Gender-neutral comparison of dental findings in the reference group and the NF1group. Findings were assigned to the 16 dental classification criteria in a side-specific manner (Identification of teeth: Number 1-8 address permanent teeth, M1-M5 deciduous teeth).

**Table 3 cont.**
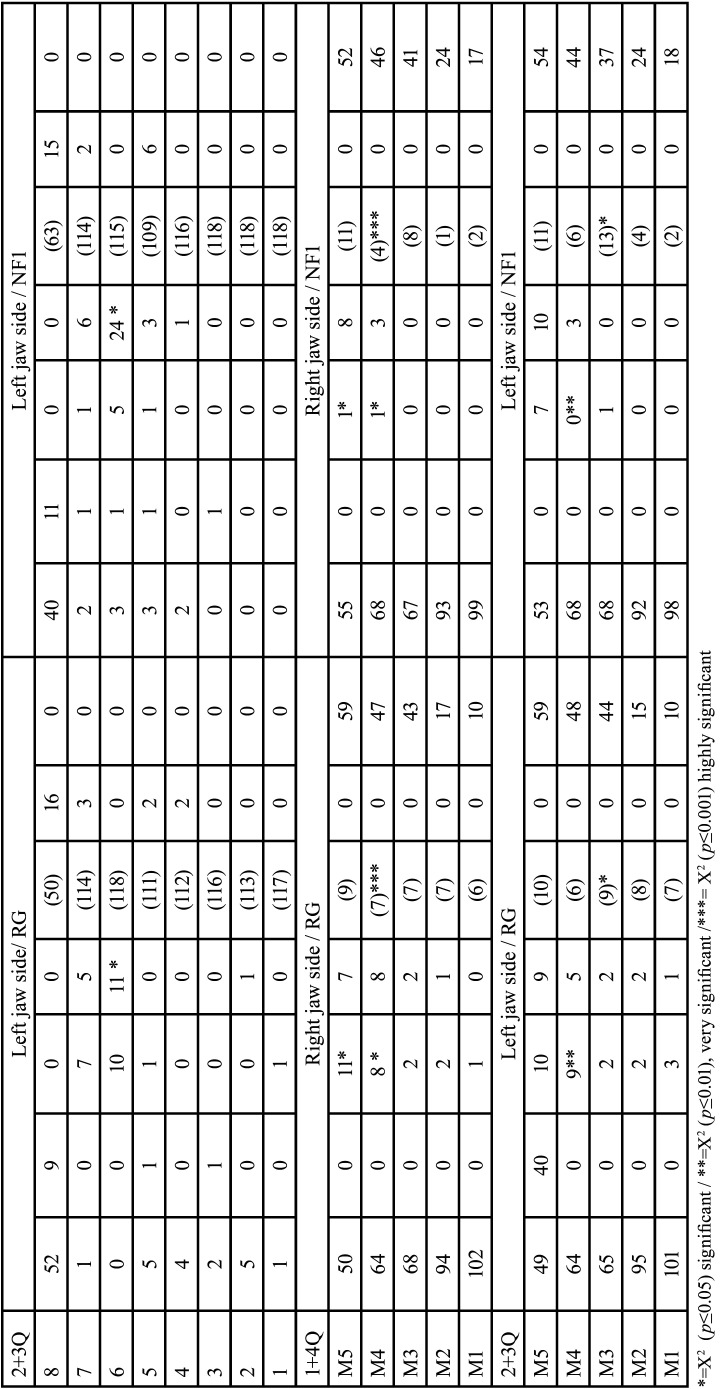
Gender-neutral comparison of dental findings in the reference group and the NF1group. Findings were assigned to the 16 dental classification criteria in a side-specific manner (Identification of teeth: Number 1-8 address permanent teeth, M1-M5 deciduous teeth).

2.1.1. Deciduous teeth

- Right jaw side comparison of deciduous teeth of both groups

Number of teeth. The number of missing teeth (1st + 4th quadrants) was high in both groups (RG: 378/590; NFG: 382/590, *p*=0.910). A high number of deciduous teeth were partially resorbed (RG: 179, NFG: 182). Accordingly, the number of teeth evaluable for assessing developmental stages was low (RG: 33, NFG: 26; *p*=0.374).

Developmental stages. The developmental stages of first deciduous molars differed significantly between both groups (*p*<0.003 (t-test), Table 4). Seven out of 36 first deciduous molars were tenth developmental stage on the right side in RG. In NFG, two out of 26 deciduous first molars on the right side were assigned to the sixth developmental stage and two out of 26 to the seventh stage. The development of the first deciduous molars was thus more advanced on the right side of the jaw on the part of the RG (Tables  Table 3, Table 4). However, it has to be considered that on the right side only eleven first primary molars of both patient groups were classified according to the growth stages. The significant difference in the development stages of deciduous first molar was thus relativized by the small number of values and the clear numerical excess of teeth (in both groups) already apically resorbed.

**Table 4:**
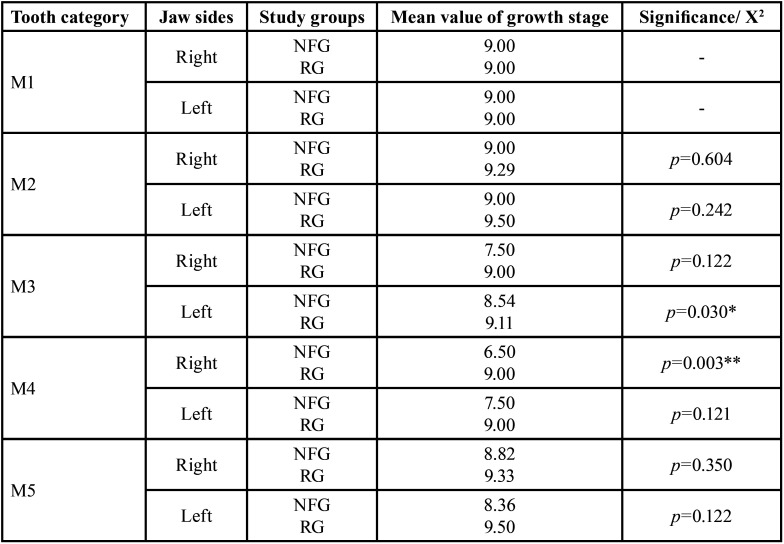
Comparison of deciduous teeth‘growth stages (mean values) independent of gender between reference group (RG) and neurofibromatosis type 1 group (NFG) / t-test, independent samples.

Tooth retention. Retained deciduous teeth were not present in the gender-specific comparison of the right jaw sides of both patient groups.

Dental Health. Eight of 54 carious deciduous first molars were present on the right side in the RG (NFG: 1/50, *p*=0.039 (Fisher)). The deciduous second molar on the right side was significantly more frequently carious in RG compared to NFG (11/68 vs. 1/63, *p*=0.012 (Fisher), Table 3,  Table 3 cont.).

- Left jaw side comparison of deciduous teeth of both study groups

Number of teeth. The number of missing teeth on the left jaw sides (2nd and 3rd quadrants) was high in both groups (RG: 374/590; NFG: 379/590, *p*=0.762). A high number of deciduous teeth were apically resorbed (RG: 176, NFG: 177). Accordingly, the number of teeth evaluable for assessing developmental stages was low (RG: 40, NFG: 34, *p*=0.840).

Developmental stages. When comparing the individual tooth categories (M1-M5) on the left side of the jaw, the development stages of the RG’s deciduous canines differed significantly from those in the NFG (*p*=0.030, (t-test)), i.e., canines of NFG developed somewhat slower than of RG (Table 4).

Tooth retention. Retained deciduous teeth were not present in left jaw sides of both patient groups.

Dental health. Caries prevalence on the left side of the deciduous first molars was significantly increased in RG (9/54) vs. NFG (0/50), (*p*=0.004 (Fisher)). There were no significant differences between the two patient groups on the left side concerning the deciduous teeth that received conservative treatment.

2.1.2. Permanent teeth

-Right jaw sides comparison

Number of teeth. Number of missing teeth did not differ significantly between both groups (67/944 vs. 54/944, *p*=0.252).

Number of missing second molars did not differ significantly (RG:1/118; NFG 2/118, *p*=0.684).

Twenty-five tooth germs were recorded in RG and 26 in NFG (*p*>0.05).

As there were no partially resorbed permanent teeth on the right side in both study groups, 852 permanent teeth of the reference group and 864 permanent teeth of the NFG remained, which were subject to the classification criteria “growth stage”, “caries”, “fillings” and “retained”.

Developmental stages. There were no significant differences in the growth stages of the permanent teeth in the sex-independent comparison of the right jaw sides of both study groups. Mean value of the growth stages (of all permanent teeth in the sex-independent comparison of the right jaw sides) was 6.68 (RG: 106.5) and 6.67 (NFG: 108.3). Findings are summarized in Tables  Table 3, Table 5.

**Table 5:**
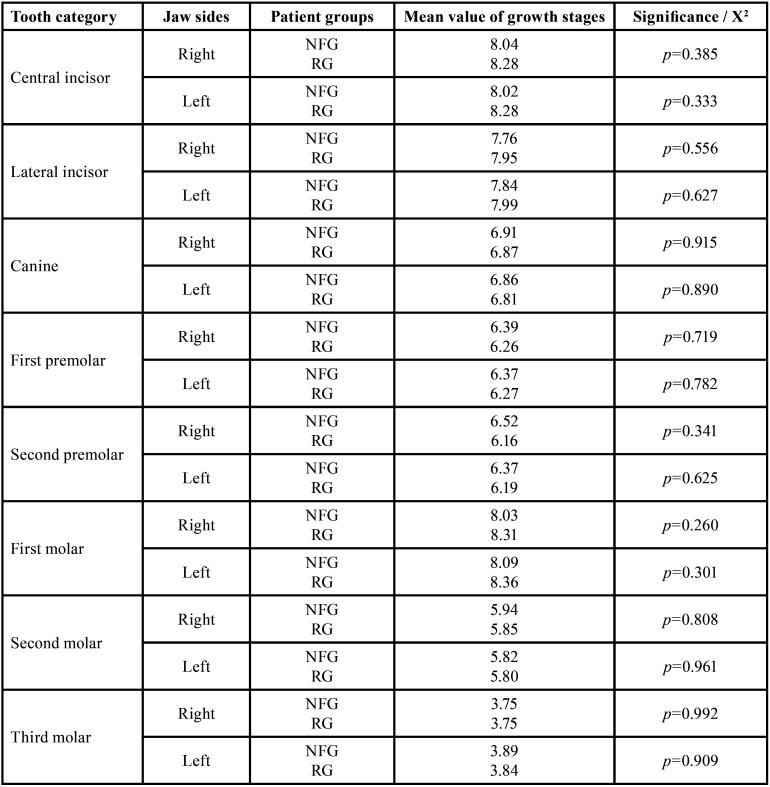
Comparison of permanent teeth‘growth stages (mean values) independent of gender between reference group (RG) and neurofibromatosis type 1 group (NFG) / t-test, independent samples.

Tooth retention. When comparing the right sides of the jaw, 15 of 864 teeth in the NF1 group were retained. In the reference group, 10 of 852 permanent teeth were retained (*p*=0,338). There were no significant differences when comparing the individual tooth categories (1-8).

Dental health. Significant differences in number of carious lesions were revealed for first molars: RG patients showed a higher number of decayed teeth (RG: 10/118, NFG: 2/118, *p*=0.036 (Fisher)).

-Left jaw side comparison of permanent teeth of both groups

Number of teeth. Number of missing teeth did not differ significantly between both study groups (RG: 70/944 vs. NFG: 50/944, *p*=0.077); number of missing second incisors (RG: 5/118 vs. NFG 0/118, *p*=0.060 (Fisher)). There were no significant differences when comparing the individual tooth categories.

Twenty-three tooth germs each were recorded comparing the left sides of the jaws in both study groups.

As there were no partially resorbed permanent teeth on the left side in both study groups, 851 teeth of the RG and 871 teeth of the NFG remained which were subject to further evaluation.

Developmental stages. The left jaw sides of both patient groups did not show any significant differences in the developmental stages (t-test). Mean value of the growth stages (of all permanent teeth in the sex-independent comparison of the left jaw sides) was 6.69 (RG: 106.38) and 6.66 (NFG: 108.88).

Tooth retention. When comparing the left sides of the jaw, 11 of 851 teeth in the reference group were retained. In the NF1 group, 15 of 871 permanent teeth were retained on the left side (*p*=0.472). The gender-independent comparison of the individual tooth categories (1-8) revealed no significant differences in retained teeth.

Dental health. An increased number of carious second molars was recorded in the RG (RG 7/114; NFG 1/114, *p*=0.066 (Fisher)). On the other hand, significantly more restored first molars on the part of the NFG were recorded (NFG 24/115; RG 11/118, *p*=0.034). From the results, it can be inferred that carious teeth were treated earlier in NF1 patients (on both sides). Findings are summarized in Tables  Table 3 and  Table 5.

2.2. Total group comparison considering sex

The evaluation of study groups considered the classification criteria of deciduous and permanent teeth, body side (jaw sides) and sex (Tables  Table 6- Table 15).

**Table 6:**
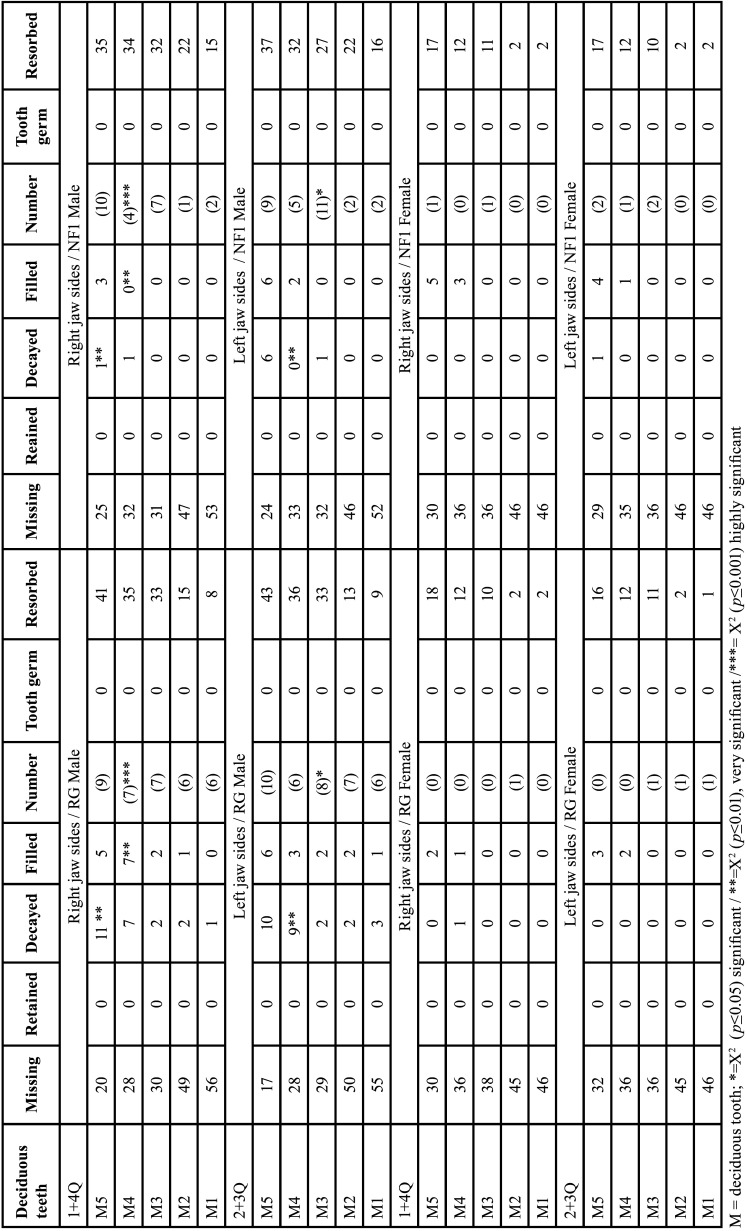
Gender-specific comparison of dental findings of deciduous teeth considering 16 dental classification criteria of both patient groups of the right jaw side and the left jaw side in each case.

**Table 7:**
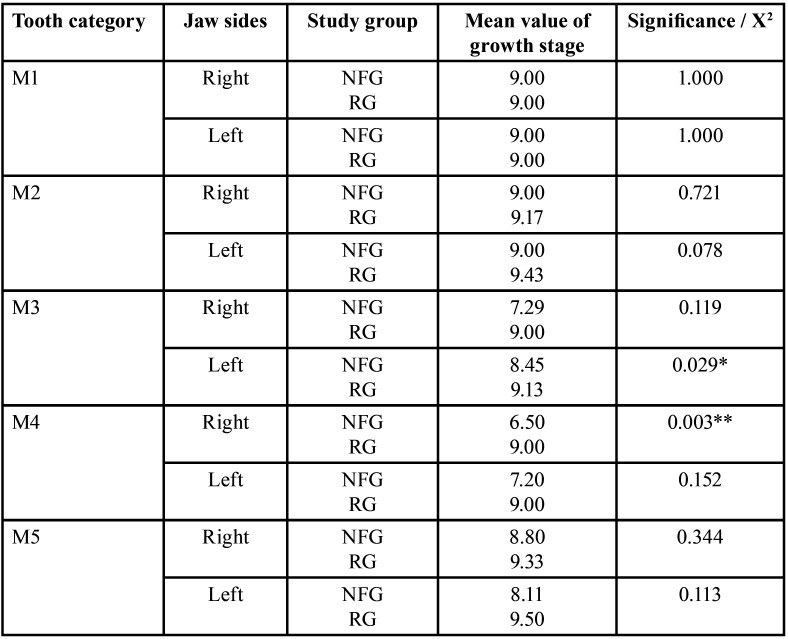
Gender-specific comparison of growth stages (mean values) per tooth category (deciduous teeth, M1-M5) between reference group (RG) and neurofibromatosis group (NFG): Males.

**Table 8:**
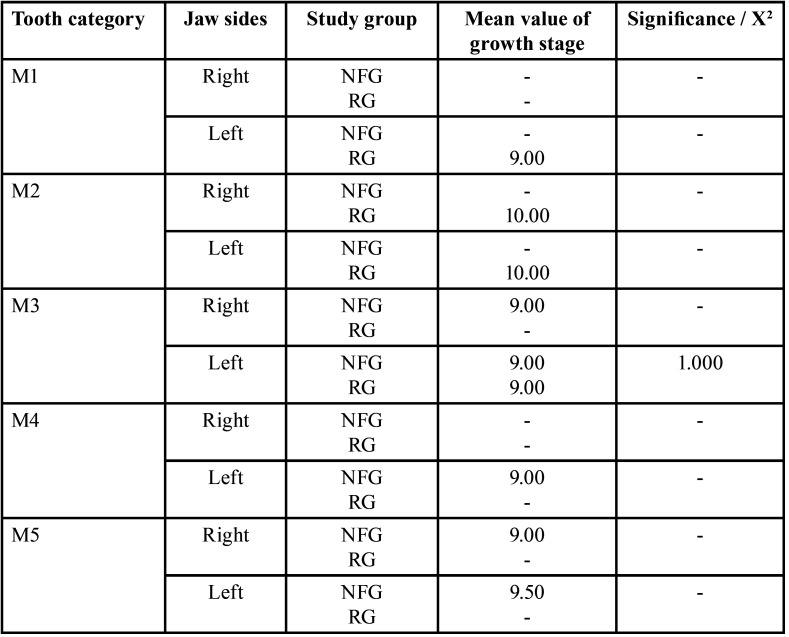
Gender-specific comparison of growth stages (mean values) per tooth category (deciduous teeth, M1-M5) between reference group (RG) and neurofibromatosis group (NFG): Females.

**Table 9:**
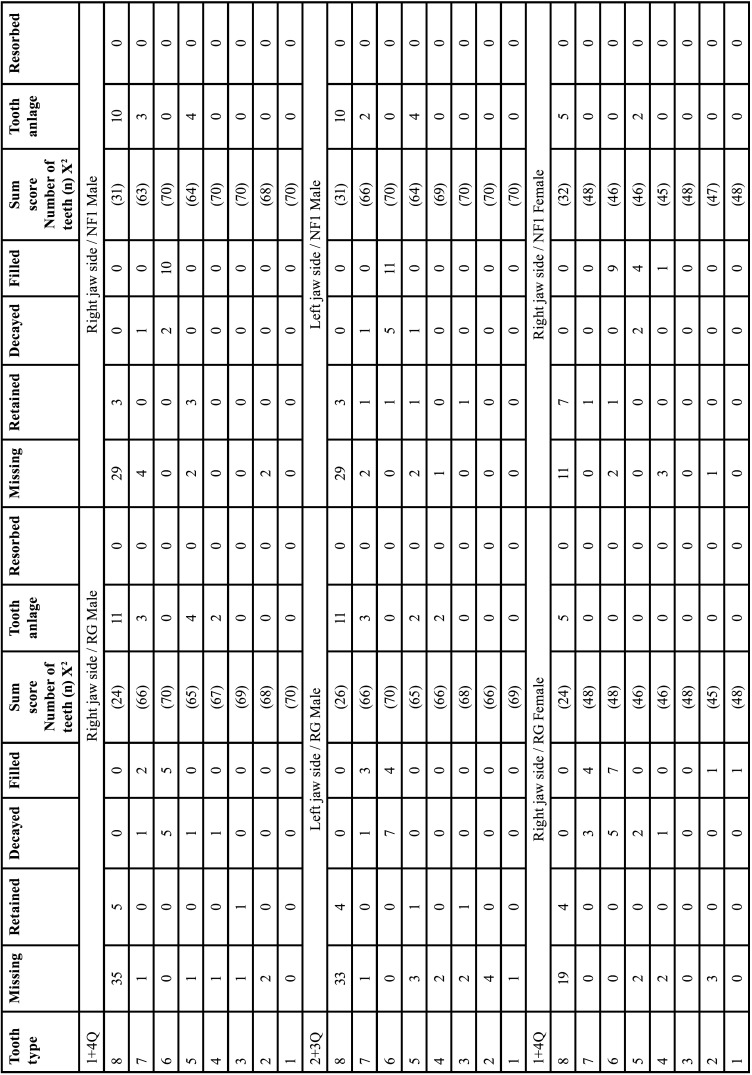
Gender-specific comparison of the number of permanent teeth, considering the 16 dental classification criteria of both patient groups of the respective right and left jaw sides.

**Table 9 cont.**
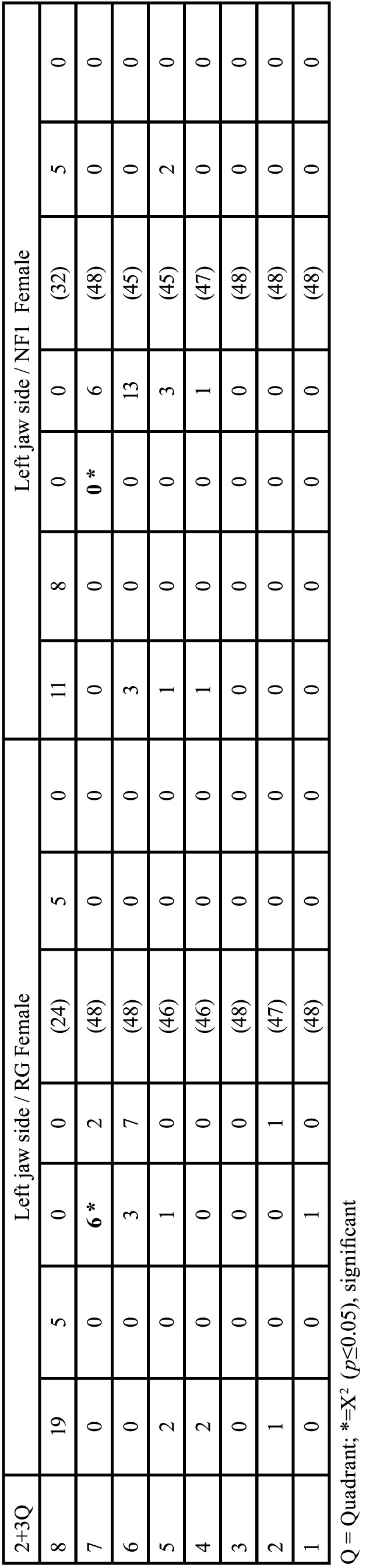
Gender-specific comparison of the number of permanent teeth, considering the 16 dental classification criteria of both patient groups of the respective right and left jaw sides.

**Table 10:**
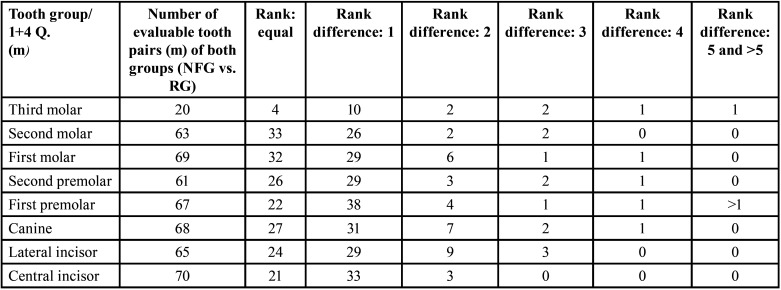
Overview of the evaluable pairs and rank differences of developmental stages of the right jaw sides between 35 male NF1 patients and 35 males (m) of the reference group (permanent teeth).

**Table 11:**
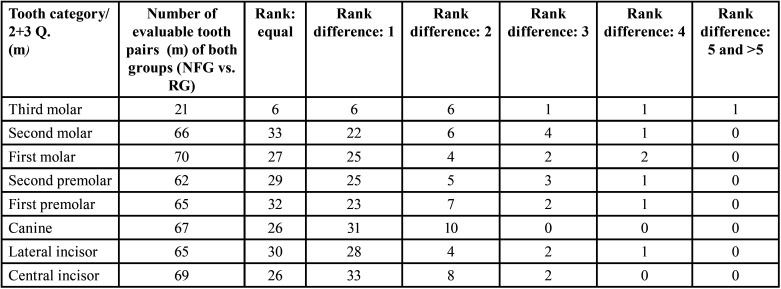
Overview of the evaluable pairs and rank differences of the developmental stages of the left jaw sides between 35 male (m) NF1 patients and 35 males of the reference group (permanent teeth).

**Table 12:**
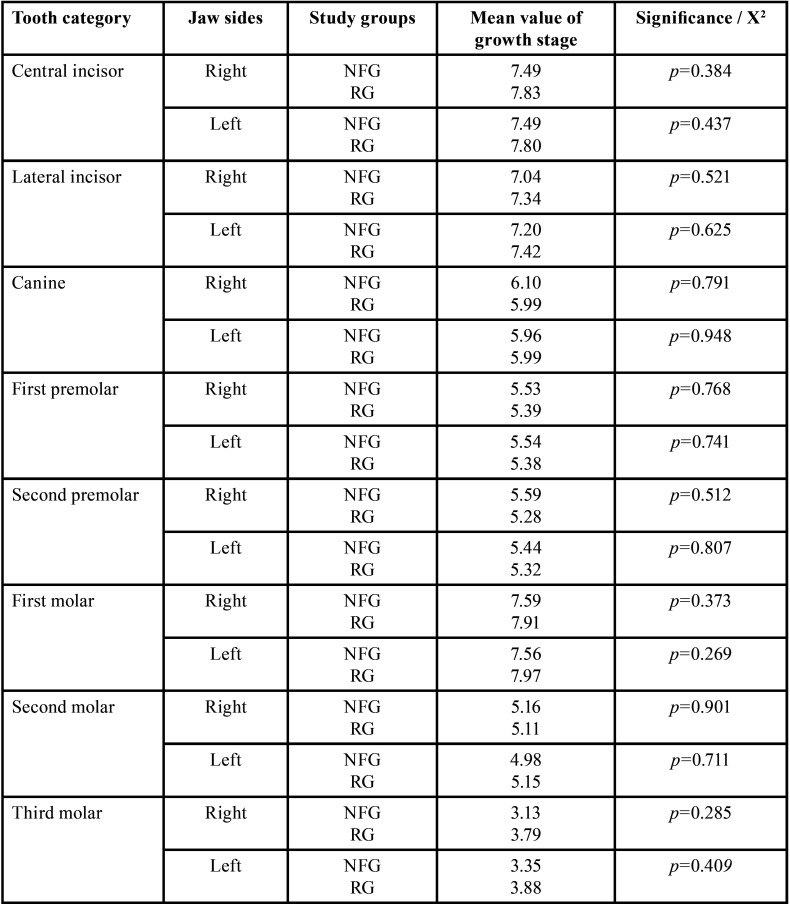
Gender-specific comparison of growth stages (mean values) per tooth category (permanent teeth) between reference group (RG) and neurofibromatosis group (NFG): Males.

**Table 13:**
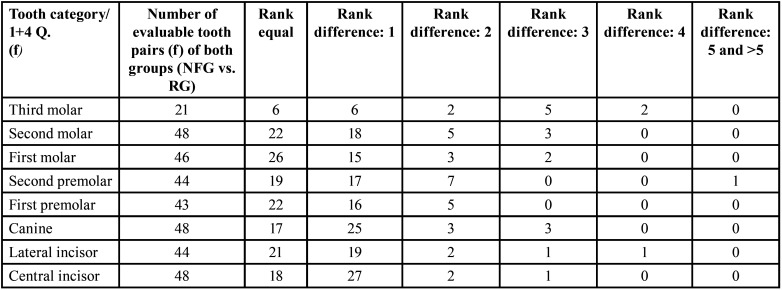
Overview of the evaluable pairs and rank differences of the developmental stages of the right jaw sides between 24 female (f) NF1 patients (NFG) and 24 females (f) of the reference group (RG) (permanent teeth).

**Table 14:**
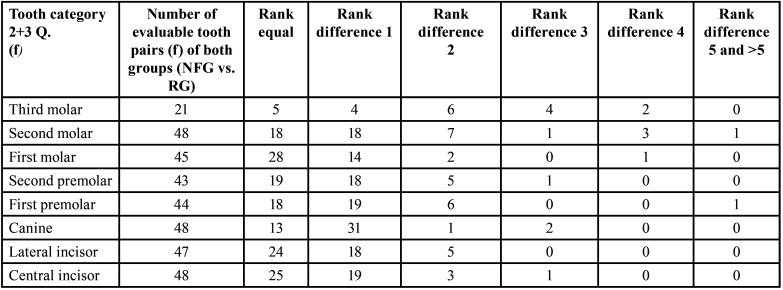
Overview of the evaluable pairs and rank differences of the developmental stages of the left jaw sides between 24 female (f) NF1 patients and 24 female patients of the reference group (permanent teeth).

**Table 15:**
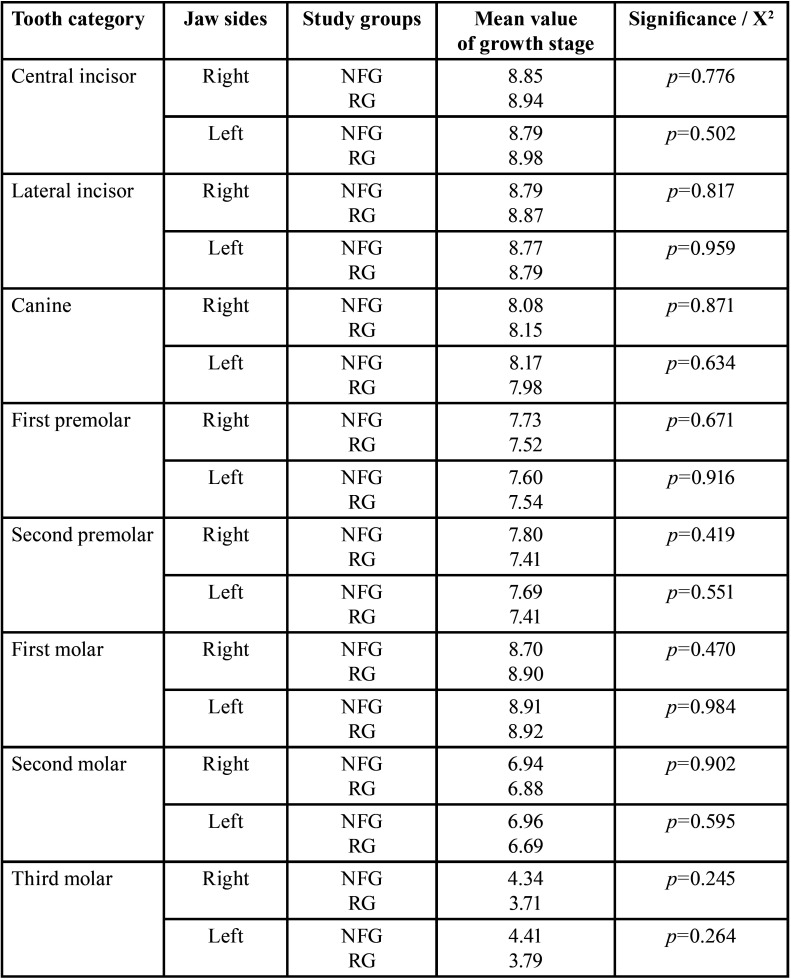
Gender-specific comparison of growth stages (mean values) per tooth category (permanent teeth) between reference group (RG) and neurofibromatosis group (NFG): Females.

2.2.1. Deciduous teeth

Of a total of 1180 expected deciduous teeth, 378 deciduous teeth were missing on the right side and 374 deciduous teeth on the left side of the RG. In the NFG, a total of 382 deciduous teeth were missing on the right side and 379 deciduous teeth on the left side (evaluable: RG: 212 right side, 216 left side; NFG: 208 right side, 211 left side). None of the values were statistically significantly different in the group comparison.

Males (Tables  Table 6, Table 7)

Right jaw sides of males

Number of teeth. Number of missing teeth did not differ significantly between both groups (RG: 183/350, NFG: 188/350, *p*=0.834; evaluable: RG: 167; NFG: 162).

Developmental stages. Number of teeth with partially resorbed roots did not differ significantly between both groups (RG: 132/167, NFG: 138/162). Since teeth with partially resorbed roots were excluded from the assessment of growth stages, 59 teeth remained to be assessed (RG: 35, NFG: 24). There was a significant difference in the developmental stages of the deciduous first molars of the males on the right side (*p*=0,003 (t-test)): Deciduous teeth developed faster in RG. Seven of the first deciduous molars fell into the tenth developmental stage on the part of the male RG. In the male NFG, two first molar teeth were assigned to the second and two to the seventh stage of development.

It is important to consider significant differences were revealed only in direct comparison of tooth entities. The number of affected teeth in relation to the total number of deciduous teeth showed no significant differences between both study groups.

The deciduous molars of the NF1 patients developed more slowly than those of the control group.

Tooth retention. All evaluable deciduous teeth had emerged to the oral cavity.

Dental health. The deciduous teeth of RG males had a significantly higher prevalence of caries on the right side of the jaw (RG: 23/167 vs. NFG: 2/162, *p*<0.001, (Fisher)).

Comparing tooth entities, deciduous second molars showed a significant increase in number of carious defects on the part of male RG (11/50 vs. 1/45 (NFG); *p*=0.012 (Fisher)). The number of carious deciduous first molars was increased in the male RG (RG: 7/42; NFG: 1/38, *p*=0.072, (Fisher)).

The number of deciduous first molars with fillings was significant higher in the RG (RG 7/42; NFG 0/38, *p*=0.017 (Fisher)).

Left jaw sides of males

Number of teeth. In total, 179 of 350 deciduous teeth of the male RG were missing on the left side (NFG: 187/359, *p*=0.735).

Developmental stages. There was no significant difference in the number of partially resorbed deciduous teeth on the left side (RG: 134/171, NFG: 134/163, *p*=0.770). There was only a significant difference in developmental stages when comparing the deciduous canines of male patients on the left jaw side (*p*=0.029). The development of deciduous canines was advanced in the RG.

Tooth retention. Retained deciduous teeth were not present on the left side of either patient group.

Dental health. Twenty-six of 171 deciduous teeth of RG were carious on the left side and seven of 163 deciduous teeth of NFG (*p*=0.003). Number of carious deciduous first molars of males was higher in RG compared to NFG (RG 9/42; NFG 0/37, *p*=0.009 (Fisher)). There were no significant differences in deciduous tooth fillings when comparing the individual tooth categories (M1-M5) of both patient groups.

Females (Tables  Table 6 and  Table 8)

Right jaw sides of females

Number of teeth. Number of missing teeth showed no statistically significant difference between both groups (RG: 195/240; NFG: 194/240, *p*=0.970). Dental findings were recorded in 45 (RG) and 46 (NFG) individuals.

Developmental stages. Number of partly resorbed roots was high in both groups (RG: 44/45; NFG: 44/46, n.s.). It follows that the number of deciduous teeth with assessable tooth development status was too small for meaningful statistical evaluation.

Tooth retention. Retained deciduous teeth were not present on the right side of either group.

Dental health. Carious lesions were rare (RG: 1/45; NFG: 0/46, *p*=0.989, (Fisher)). Number of filled teeth was higher in NFG (RG: 3/45, NFG 8/46, *p*=0.210 (Fisher)).

Left jaw sides of females

Number of teeth. Number of missing deciduous teeth did not differ significantly between both groups (RG: 195/240 vs. NFG 192/240 teeth). The number of partially resorbed deciduous teeth did not differ significantly on the left side of the jaw (RG: 42/240 teeth vs. 43/240 teeth, *p*=0.910).

Developmental stages. The tooth change of the female groups was clearly more advanced compared to males. Therefore, developmental stages of deciduous dentition could only be assessed in individual cases. In the female patients, only three deciduous teeth of the RG and five deciduous teeth of the NFG of 350 teeth each fell within the classification criteria of the developmental stages on the left side of the jaw.

Tooth retention. Retained deciduous teeth were not present in either female patient group.

Dental health. There were also no significant differences in caries distribution and number of fillings between the two female patient groups on the left side.

In summary, the comparisons show the symmetry the deciduous dentition of both study groups, as well as the acceleration of the dentition in girls compared to boys, which can be registered independently of group affiliation. Carious teeth of deciduous dentition are more common in RG boys. There are no analogous findings in girls.

2.2.2. Permanent teeth

944 permanent teeth per jaw side and study group were assessed. Sixty-seven permanent teeth of the right jaw side of the RG (7.1%) and 54 permanent teeth of the NFG were missing (5.7%, *p*=0.252). On the left side, 70 permanent teeth of 944 teeth of the RG were missing (7.4%). In the NFG, 50 of 944 teeth were missing (5.3%, *p*=0.077) (Table 9, 9 cont.).

Considering the factor ‘sex’ in both groups, on the right side 41 of 560 permanent teeth were missing in males and 26 of 384 teeth missing in females of the RG (*p*=0.763) (NFG: males 37/560; females 17/384, *p*=0.180). On the left side, missing permanent teeth were recorded in RG (46/560 (males); 24/384 (females), *p*=0.293) and NFG (34/560 (males); 16/384 (females), (*p*=0.223).

Tooth germs were recorded on the right side in 20 cases (males) and 5 cases (females) of RG (NFG: 17 (males), 7 (females)). On the left side, 18 (males) and 5 (females) tooth germs were recorded in RG (NFG: 16 (males), 7 (females), n.s.).

In the NFG, 506 permanent teeth remained in male patients and 360 in the female patients. Considering the missing teeth and tooth germs, 496 permanent teeth remained on the left side of the males in the RG and 355 permanent teeth in the females in the RG (NFG: 510 (males) and 361 (females)) for further classification.

Males

- Right jaw sides of males (Table 10)

Number of teeth. In 35 male patients, a total of 1120 teeth/tooth positions were to be evaluated (560 teeth per jaw side). In total, 41 permanent teeth were missing on the right side of the males in the RG (7.32%) and 37/560 (6.61%) in the NFG (*p*=0.661). Difference in number of tooth germs was minimal (RG: 20; NFG: 17, *p*=0.612). The number of third molars was higher in NFG (RG: 24/70; NFG: 31/70, n.s.).

Developmental stages. A total of 499 and 506 permanent right-sided teeth were evaluable for assessing dental developmental stages in RG and NFG, resp. Comparison of the growth stages of the individual tooth categories (central incisors to third molars) revealed no significant difference on the right side in males of both groups. There were no significant differences of the developmental stages considering the tooth categories of RG and NFG males. There was no evidence of an effect of disease on the development stages of third molars.

Tooth retention. On the right side, the number of impacted teeth of males in both groups did not differ significantly. Six of 499 permanent teeth of the male patients in the RG were retained. The male patients of the NFG were affected by six of 506 teeth on the right side of the jaws (*p*=0.981).

Dental health. RG males had more carious lesions on the right side compared with NFG males (RG: 8/499, NFG: 3/506, *p*=0.143 (Fisher)). The number of restored teeth was higher in NFG males (10/506) than in RG males (7/499, *p*=0.488).

- Left jaw sides of males (Table 11)

Number of teeth. Total number of missing teeth of the left jaw sides did not differ significantly between both groups RG: 46/560; NFG: 35/560, *p*=0.238). Number of missing lateral incisors did not differ significantly (4/70 (RG) vs. 0/70 (NFG), *p*=0.129 (Fisher)).On the left side, 18 tooth germs were present in the male RG (NFG: 16). A total of 496 permanent teeth of the male RG and 510 permanent teeth of the NFG remained evaluable on the left side.

Developmental stages. Comparison of the growth stages of the individual tooth categories revealed no significant differences on the left side in the males of both groups (Table 12).

Tooth retention. Six of 496 permanent teeth of the male patients in the RG were retained. The male patients of the NFG were affected by seven of 510 teeth on the left side of the jaws (*p*=0.821).

Dental health. The number of decayed teeth did not differ significantly in males (RG: 8/496; NFG: 7/510, *p*=0.757). There were no significant differences in number of restored teeth (RG: 7/496; NFG: 11/510, *p*=0.381).

The individual comparisons of the tooth categories (central incisors to third molars) with respect to the criteria “decayed” and “filled” also revealed no significant differences in the left jaw sides in males of both groups.

-Right jaw side of females (Table 13)

Number of teeth. On the right jaw sides, more teeth were missing in female RG compared to female NFG (RG: 26/384; NFG: 17/384, *p*=0.182). Number of tooth germs was low in both groups (RG: 5; NFG: 7, *p*=0.596). There were no resorbed roots of permanent teeth in both groups (evaluable number of teeth: RG: 353, NFG: 360).

Developmental stages. The developmental stages showed no significant differences in females of both groups when comparing the respective tooth developmental scores (central incisors to third molars) on the right side.

Tooth retention. Four of 353 permanent teeth of the female patients in the RG were retained. In the female patients of the NFG, retention affected nine of 360 of the right sides of the jaw.

Dental health. In the female RG, carious permanent teeth were significantly more frequently recorded (11/353) compared to NFG (2/360), (*p*=0.021 (Fisher)). When comparing the first molars of the female patients of both study groups, the difference in carious teeth on the right side was not significant (RG: 5/48; NFG: 0/46, *p*=0.059 (Fisher)).

In females of RG, no fillings were present on the right side of 46 second premolars, compared to four of 46 second premolars in the NF1 group, (*p*=0.118 (Fisher)).

- Left jaw side of females (Table 14)

Number of teeth. Number of missing teeth on the left side of the jaws of the females were more frequent in the RG (24/384 teeth; NF1=16/384, *p*=0.218).

Developmental stages. The developmental stages showed no significant differences in females of both groups when comparing the respective tooth developmental scores (central incisors to third molars) on the left side (*p*=0.435 (Fisher), Table 15).

Tooth retention. Five of 355 permanent teeth of the female patients in the RG were retained. In the female patients of the NFG, it affected eight of 361 of the left sides of the jaw. Individual comparisons of the tooth categories (central incisors to third molars) did not reveal any significant differences.

Dental health. The difference in number of decayed teeth was significant on the left side for the second molars (*p*<0.001). Caries was more frequent on the left side in the RG (11/355) than in the NFG (0/361).

In summary, both groups showed almost no differences of dental parameters when gender was considered. However, dental health was somewhat better maintained in NFG.

3. FPNF vs. DNF

NFG (n=59) was subdivided according to present (n=34 (57.63%), FPNF) or absent (n=25 (42.37%), DNF) FPNF. Differences in dental findings of deciduous teeth were not significant comparing FPNF vs. DNF. Only findings of permanent dentition are further detailed. The jaw sides were first compared by subgroup affiliation without considering the unilateral manifest factor ‘facial PNF side’ ( Table 16- Table 18).

**Table 16:**
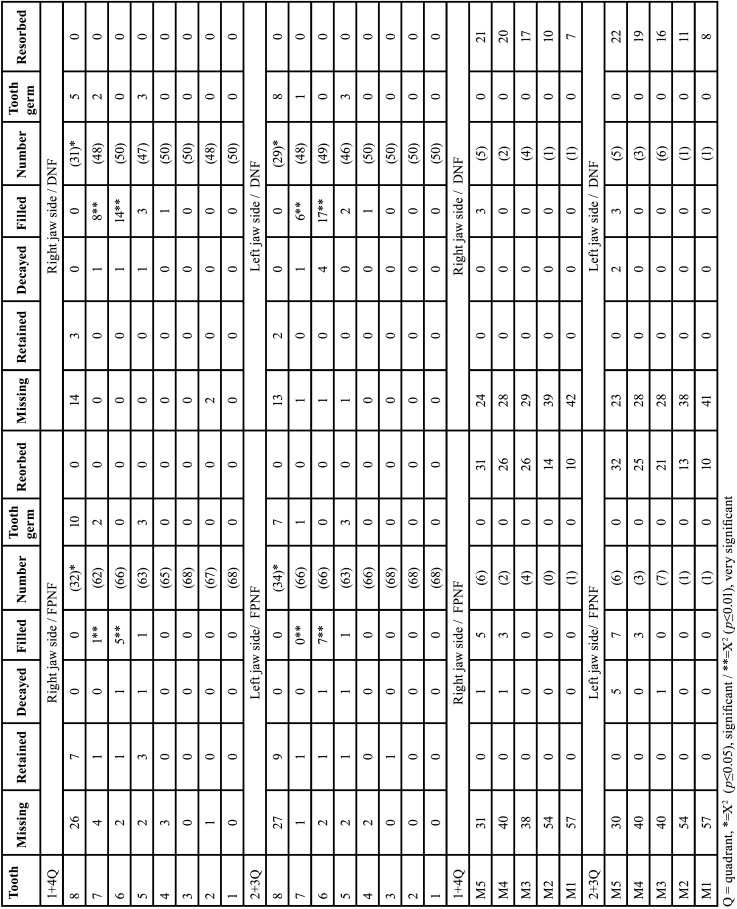
Side-by-side comparison of dental findings within the NF1 group. NF1 patients are distinguished by presence (FPNF group) or absence (DNF group) of FPNF in permanent (upper part of table) and deciduous (lower part of table) dentition (Identification of teeth: Number 1-8 address permanent teeth, M1-M5 deciduous teeth).

**Table 17:**
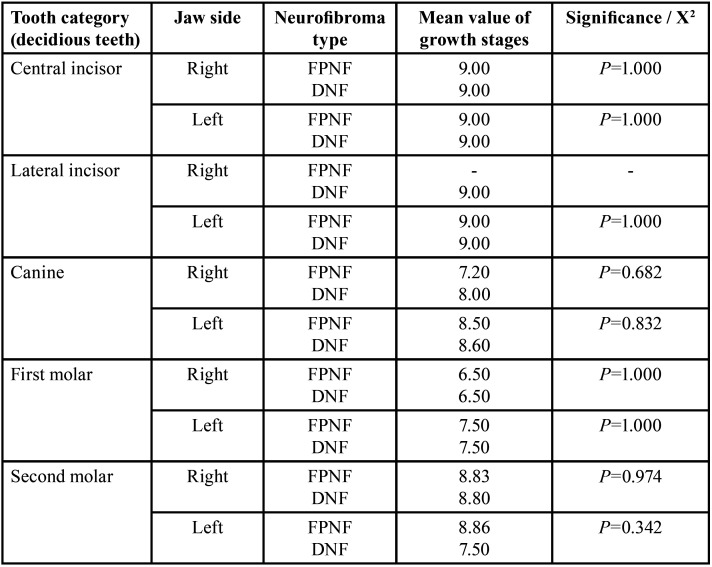
Differences of jaw-side specific growth stages within the NF1 group: FPNF vs. DNF (deciduous teeth, t-test, independent samples).

**Table 18:**
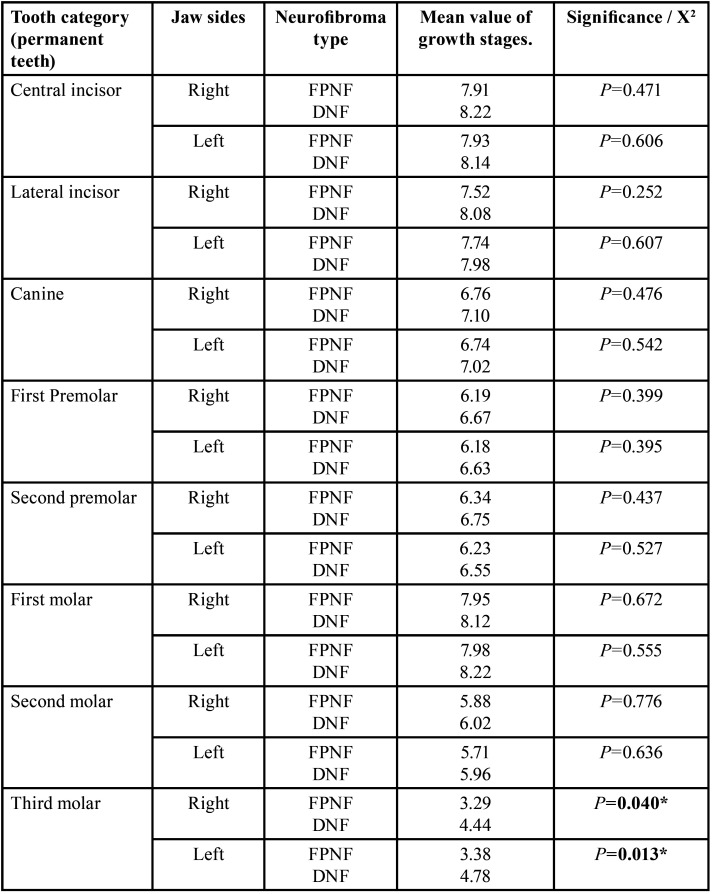
Differences of jaw-side specific growth stages within the NF1 group: FPNF vs. DNF (permanent teeth, t-test, independent samples).

- FPNF vs. DNF, right jaw sides

Number of teeth. The number of missing third molars was high and did not differ between both NF subgroups (FPNF: 26/68; DNF: 14/50, *p*=0.412).

Developmental stages. The comparison of the third molars on the right jaw in patients with FPNF or DNF showed a significant difference in growth stages (*p*=0.040). On mean, the third molars of patients with DNF (mean 4.44) were more developed than those with FPNF (mean 3.29).

Tooth retention. Twelve of 491 permanent teeth of the right side of the jaw in patients with FPNF were retained (DNF patients 3/374, *p*=0.112, (Fisher)). The individual comparison of the tooth categories (central incisors to third molars) did not show any significant differences. The number of retained teeth was increased in the patients with FPNF. A correlation to the affected jaw sides and the associated eruption obstruction due to a tumor could not be proven subsequently.

Dental health. The number of restored second molars on the right side of the jaw was significantly different between both NFG (FPNF: 1/62; DNF: 8/48, *p*=0,013 (Fisher)). The first molar of the right side of the jaw showed a significant difference in number of fillings when comparing the NFG groups (FPNF: 5/66; DNF: 14/50, *p*=0,013).

- FPNF vs. DNF, left jaw sides

Number of teeth. The number of missing third molars was 34/68 (FPNF) and 13/50 (DNF) (*p*=0.079). Each one second molar was missing in both subgroups.

Developmental stages. The third molars on the left jaw in patients with FPNF or DNF showed a significant difference in growth stages (*p*=0.013 (t-test)). On mean, the third molars of patients with DNF (mean: 4.78) were more developed than those with FPNF (mean: 3.38).

Tooth retention. 13 of 499 permanent teeth on the left side of the jaw in patients with FPNF were retained. In the patients with DNF, two of 372 permanent teeth were affected (*p*=0.032 (Fisher)). The number of retained teeth in the patients with FPNF was overall significantly increased compared to patients with a DNF (FPNF: 13/499; DNF 2/372, *p*=0.032). Again, there were no significant differences when comparing the individual tooth categories (central incisors to third molars) concerning this parameter. A connection to the affected jaw sides and the associated eruption obstruction due to a tumor could not be proven subsequently.

Dental health. There was a significant difference in the number of second molars that had received restorations (FPNF: 0/66; DNF: 6/48, *p*=0.007 (Fisher)). The first molars of patients with a DNF also had more restorations than FPNF affected individuals (7/68 FPNF vs. 17/49 DNF, *p*=0.009 (Fisher)).

Tooth eruption and exfoliation of deciduous teeth do not appear to be influenced by the presence of oral neurofibroma according to the data of the study. In contrast, well-documented data are available demonstrating PNF-associated tooth retention or delayed emergence to the oral cavity of permanent dentition, especially in the area of teeth without precursors.

4. FPNF group: intra-individual comparison of dental findings in affected vs. unaffected jaw sides.

In this comparison of findings, the order scheme is not the side-by-side comparison according to the right-left body scheme but is determined according to affected vs. unaffected side. The growth stages were not tabulated in the first dentition due to the high number of missing deciduous teeth and partially absorbed deciduous tooth roots. Tooth development stages and tooth replacement showed no side difference in deciduous and permanent dentition in NF1 patients with FPNF (n=34). Of 34 patients with FPNF, 18 patients were affected on the left sides and 16 on the right sides of the jaws.

Affected vs. unaffected side

Number of teeth. The comparison of the number of missing third molars of the affected sides and unaffected sides was not significant (Affected: 26/68, unaffected: 27/68, *p*=0.907). The number of tooth germs is high for third molars due to the age structure of the patient group (Affected: 9/42; unaffected: 8/41, *p*=0.861). All other tooth comparisons showed a very small number of tooth germs and missing teeth.

Developmental stages. The upper and the lower jaw are considered separately. The stages of development showed no significant differences between the effected and unaffected jaw sides. Even the individual tooth entity comparisons revealed no relevant abnormalities.

Tooth retention. The individual comparison of the tooth categories (central incisors to third molars) did not show any significant differences in retention. On the affected jaw sides, a total of eleven out of 492 teeth were retained. On the unaffected jaw sides, 13 out of 499 teeth were retained (Affected: 11/492; unaffected: 13/499, *p*=0.712).

Dental health. There were no significant differences in caries and fillings between the affected and unaffected sides of the jaw. In total, there was one more tooth filled and four more teeth that had received restorations (Fillings: affected: 2/492; unaffected: 3/499, *p*=0.998) (Caries: affected: 5/492; unaffected: 9/499, *p*=0.421). There were also no differences when comparing individual permanent teeth.

## Discussion

In the present study, we investigated whether mixed dentition is affected in NF1 patients considering the impact of NF1-associated orofacial tumors. Overall, the presented results show that tooth development in NF1 patients is in line with the expected dentition of the general population. This study addresses for the first time the influence of the type of facial peripheral nerve sheath tumors of NF1 patients on tooth development. It is known that FPNF can be associated with significant jaw deformities and tooth position anomalies (21,22). Furthermore, it was determined whether there are differences in tooth development in the intra-individual side-by-side comparison of the unilaterally developing FPNF.

There are some reports on dental growth stages and health status in children and adolescents with NF1 (7-12,16,21,23-33). The presented investigations on symmetry of dental development and health suggests that the development and change of the teeth of the first and second dentition in NF1 patients does not differ considerably from the general population. Neither has the autosomal dominant inheriTable disease a general effect on tooth development, nor can tumor-specific effects of facial plexiform neurofibromas on tooth development be demonstrated.

The present study does not confirm a previously reported finding that deciduous dentition occurs earlier in NF1 patients than in the general population (7). This conclusion could not be confirmed in another study (8). However, the above mentioned two studies only used oral inspections as the basis for calculations (and the study cohorts varied in group size, population recruitment, and scoring method). In contrast, only dental X-ray findings were used for the evaluation here. The group size of the presented study is like that of the Finnish working group (8). Jääsaarie *et al*. (8) described an advanced dental age in girls with NF1 compared with Finnish norms, whereas the dental age of both sexes with NF1 showed no significant differences when compared with dentition diagrams of Finnish population. The study results presented suggest that there are no significant differences in tooth development and tooth change between NF1 patients and the normal population in deciduous teeth.

However, the FPNF has an association with the developmental delay of some permanent teeth on the tumor side. The effect is evident in wisdom teeth. This observation is interesting because these teeth appear and develop as the last entity on the timeline of tooth development. Causes for the developmental delay cannot be deduced from the radiological findings. However, the frequent finding of more distal position of premolar and molar teeth topographically related to a FPNF is conspicuous (32). The impaired mesial migration of the teeth during jaw and tooth development could cause the developmental delay of the wisdom teeth. On the other hand, the oral PNF is an effective barrier to tooth eruption. However, in this study the deciduous teeth emerge in the oral portion of FPNF without a lateral difference to the unaffected side. It is unknown whether the osseous branches of the inferior alveolar nerve running to the dental apices and alveolar bone on the FPNF side are also tumorous. Based on the radiological findings, it can be cautiously assumed that development and eruption of first dentition are not delayed in NF1 children, even in patients with extensive FPNF. In contrast, delayed visibility of the dental crown in the oral cavity or impaired eruption of permanent teeth related to FPNF invaded oral mucosa is well documented (26).

The present findings seem worth sharing because NF1 is characterized as a histogenesis control gene (6). Several publications emphasize that many NF1-associated malformations and tumors can be interpreted as consequences of disturbances of specified neural crest (NC) derivatives (1-6). NC derivatives are constitutive cells of teeth and craniofacial bones.

In addition to the PNF-associated delayed tooth eruption, there is often limited mesial drift of the replacement teeth, here primarily the premolars. However, greater impairments of mesial migration in connection with PNF are observed in molars, i.e., teeth without predecessors (12,21). Apparently, impairment or even the loss of organ function due to tooth retention has not necessarily lasting effect on organ development as such. Significant differences in tooth development in the symmetry comparison of affected and unaffected sides of the FPNF patients were not registered in frontal teeth. Apparently, in cases of proven oral PNF, the mesial migration and eruption of the teeth, especially of permanent molar teeth, may be restricted to distal areas of the alveolar process on the side of the PNF, while the development of this tooth group is complete with short time delay in completion of root development.

Differences in dental health between both study groups as well as within the NFG, and here in particular differentiated according to the side of the FPNF, are probably the result of the oral tumor growth and associated functional deficits, e.g., facial palsy, both affecting conditions of oral hygiene. In addition, the quality and utilization of public health care undoubtedly has an impact on dental health status in NF1 and may also be partly responsible for the fact that the DMFT index data for NF1 patients show national differences (9-12). For our own patients, the findings suggest the general conclusion that NF1-affected individuals in childhood and adolescence exhibit less frequent caries and more frequent fillings. The FPNF likely has a site-specific effect on dental health. The verification of the results on a larger comparison group is to be aimed at. Tucker *et al*. (10) described increased caries prevalence in patients with NF1. In a recent study of 179 NF1 patients and controls, DMFT values were elevated in NF1 patients (16). However, there was a higher rate of caries in patients in the RG (16). Previously, Visnapuu *et al*. (11) described better dental health status in individuals with NF1. In the Finnish study, the number of carious teeth was increased on the part of the RG, which is consistent with the present study on children and adolescents with NF1. Interpreting the low total number of decayed and restored teeth in both study groups in this study, their low average age and thus the teeth’ relatively short exposure time of the teeth to the oral cavity must be considered. Decayed teeth in NF1 patients are apparently the result of individual hygiene standards, indicators of the quality of care provided by the respective health care system and may be influenced by a FPNF (tumor volume, periodontal growth, functional impairment of facial muscles) and not by the genetic disease as such. The obvious argument of the possibly incomplete detection of carious teeth by the exclusively radiological study applies to both study groups and therefore does not influence the comparability of the results.

Limitations of study. The source of imaging error (OPG) due to incorrect positioning of the patient in the device or due to possible skeletal deformation in NF1 patients must be considered and cannot be excluded. The influence of imaging errors due to incorrect positioning, in addition to the known technical deficiencies of the OPG, highlights the limited interpretation accuracy of the spatial dental relationship due the trough-shaped focus of the x-rays to generate the panoramic image (34). From experience with previous OPG evaluations of NF1 patients, the limited representation of bone deformity related to tooth position is particularly true for patients who have developed FPNF affecting large areas of the second and third branch of trigeminal nerve (16).

Dental findings such as missing teeth have been described several times as oral manifestations in NF1 (21,23). In this context, the finding ‘missing’ second molars should be emphasized, because in a previous investigation missing second molar in the mandible was detected four times, and this was published as another dental characteristic of the FPNF patient (21). This finding could not be confirmed in this study. In this study, since evaluation relying only on OPGs could not distinguish between tooth aplasia and extracted tooth, all absent teeth were considered ‘missing’. The presented result does not contradict an NF1-associated influence on numerical aberrations of the permanent dentition in NF1, e.g., aplasia of permanent teeth. Obviously, supernumerary teeth are to be expected in NF1, preferentially affect the premolar/molar region, and reach a prevalence that is unequivocally higher than expected in the normal population (16). Molar teeth are particularly affected (wisdom teeth), which in several cases are not yet to be expected in the study group investigated here. Therefore, changes in the number of teeth in NF1 may be registered, which could not be recorded here due to the conditions of the study group (mixed dentition, young age, radiological examination only). Therefore, further studies should be designed to specify the dental status of adult NF1 patients.

## Conclusions

When diagnosing the disease NF1, both dental health and dental development should be considered in the affected individual. The assessment of oral hygiene and health are part of the spectrum of systematic care for NF1 patients at medical centers that have been or are being set up to care for this patient group (35).

Subtly deviations in the growth stages in children and adolescents with NF1 may represent possible first signs for a diagnosis. However, radiological findings of dentition are rarely recorded in childhood, and, in view of radiation hygiene, radiography should only be performed if there is a justifiable indication. The results of the side-specific single-tooth observations of the growth stages were largely similar in both study groups in this study: PNF has an impact on bone development but does not necessarily interfere with dental development. If an effect is present, the developmental delay in time is small in teeth related to oral PNF. However, positioning, emergence and occlusal contact of teeth may be severely influenced by oral PNF. The significant differences in growth stages of the deciduous teeth indicate advanced tooth development in patients of the RG. The long development period of permanent teeth could be important for the developmental delay of the wisdom teeth in the FPNF region. Childhood and early adolescence are generally the strongest growth phase of FPNF. However, the differences in dental growth stages of age and sex-matched RG and NFG are small and identify differences between narrow developmental stages. Results should be interpreted with caution due to the small number of teeth evaluable for evaluation. Indeed, the few and discreet differences in growth stages of deciduous and permanent teeth in children and adolescents of NF1 patients and RG suggest that differences have no practical meaning and individual tooth development as such is not affected by NF1. In contrast, the impacted premolars and molars in FPNF represent a challenge to dental therapy, often associated with severe deformations of the jaw on the tumor side. Great attention should be paid to oral health of NF1 patients in order not to further burden of affected individuals.

## References

[1] Ferner RE, Gutmann DH (2013). Neurofibromatosis type 1 (NF1): diagnosis and management. Handb Clin Neurol.

[2] Legius E, Messiaen L, Wolkenstein P, Pancza P, Avery RA, Berman Y (2021). Revised diagnostic criteria for neurofibromatosis type 1 and Legius syndrome: an international consensus recommendation. Genet Med.

[3] Belakhoua SM, Rodriguez FJ (2021). Diagnostic Pathology of Tumors of Peripheral Nerve. Neurosurgery.

[4] Bergoug M, Doudeau M, Godin F, Mosrin C, Vallée B, Bénédetti H (2020). Neurofibromin structure, functions and regulation. Cells.

[5] Gutmann DH, Ferner RE, Listernick RH, Korf BR, Wolters PL, Johnson KJ (2017). Neurofibromatosis type 1. Nat Rev Dis Primers.

[6] Riccardi VM (2010). Neurofibromatosis type 1 is a disorder of dysplasia: the importance of distinguishing features, consequences, and complications. Birth Defects Res A Clin Mol Teratol.

[7] Lammert M, Friedrich RE, Friedman JM, Mautner VF, Tucker T (2007). Early primary tooth eruption in neurofibromatosis 1 individuals. Eur J Oral Sci.

[8] Jääsaari P, Visnapuu V, Nyström M, Peltonen S, Peltonen J, Happonen RP (2012). Dental age in patients with neurofibromatosis 1. Eur J Oral Sci.

[9] Tsang ES, Birch P, Friedman JM, Johnston D, Tucker T, Armstrong L (2010). Prevalence of dental caries in children with neurofibromatosis 1. Clin Oral Investig.

[10] Tucker T, Birch P, Savoy DM, Friedman JM (2007). Increased dental caries in people with neurofibromatosis 1. Clin Genet.

[11] Visnapuu V, Pienihäkkinen K, Peltonen S, Happonen RP, Peltonen J (2011). Neurofibromatosis 1 and dental caries. Clin Oral Investig.

[12] Friedrich RE, Reul A (2018). Decayed, missing, and restored teeth in patients with Neurofibromatosis Type 1. J Clin Exp Dent.

[13] Friedrich RE, Schön M (2023). Scoring system for assessing the symmetry of tooth development in mixed dentition. J Clin Exp Dent.

[14] Friedrich RE, Ulbricht C, von Maydell LA, Scheuer HA (2005). Identification of developmental stages of wisdom teeth on orthopantomograms of adolescents and young adults as an aid for forensic-odontological age-estimations: predictive values for the chronological age of 18 years. Arch Kriminol.

[15] Friedrich RE, Ulbricht C, Baronesse von Maydell LA (2003). The influence of wisdom tooth impaction on root formation. Ann Anat.

[16] Friedrich RE, Reul A (2017). Supernumerary molars and wisdom tooth shape alterations in patients with neurofibromatosis type 1. J Oral Maxillofac Res.

[17] Gleiser I, Hunt EE Jr (1955). The permanent mandibular first molar: its calcification, eruption and decay. Am J Phys Anthropol.

[18] Köhler S, Schmelzle R, Loitz C, Püschel K (1994). [Development of wisdom teeth as a criterion of age determination]. Ann Anat.

[19] Viohl J (1966). Dokumentation mit Maschinenlochkarten in der konservierenden Zahnheilkunde an Universitatskliniken. [Documentation with punched cards in conservative dentistry in university clinics]. Dtsch Zahn Mund Kieferheilkd.

[20] Jiang C, McKay RM, Lee SY, Romo CG, Blakeley JO, Haniffa M (2023). Cutaneous neurofibroma heterogeneity: factors that influence tumor burden in neurofibromatosis type 1. J Invest Dermatol.

[21] Friedrich RE, Giese M, Schmelzle R, Mautner VF, Scheuer HA (2003). Jaw malformations plus displacement and numerical aberrations of teeth in neurofibromatosis type 1: a descriptive analysis of 48 patients based on panoramic radiographs and oral findings. J Craniomaxillofac Surg.

[22] Friedrich RE, Reul A (2018). A combination of skeletal deformations of the dorsal mandible and temporomandibular region detected in orthopantomograms of patients with neurofibromatosis type 1 indicates an associated ipsilateral plexiform neurofibroma. J Craniomaxillofac Surg.

[23] Visnapuu V, Peltonen S, Alivuotila L, Happonen RP, Peltonen J (2018). Craniofacial and oral alterations in patients with Neurofibromatosis 1. Orphanet J Rare Dis.

[24] Clamors J (1972). [Preventive prosthetics for a patient with neurofibromatosa localisata Recklinghausen]. Quintessenz.

[25] Ey-Chmielewska H, Sobolewska E, Fraczak B (2007). Description of prosthetic treatment in case of neurofibromatosis in the course of Recklinghausen disease. Case course. Ann Acad Med Stetin.

[26] Kim KH, Lee DW, Lee ST (2021). Delayed tooth eruption due to gingival neurofibroma in two children with neurofibromatosis 1. J Paediatr Child Health.

[27] Wotjiuk F, Hyon I, Dajean-Trutaud S, Badran Z, Prud'homme T (2019). Dental management of neurofibromatosis Type 1: A Case Report and Literature Review. Int J Clin Pediatr Dent.

[28] Thota E, Veeravalli JJ, Manchala SK, Lakkepuram BP, Kodapaneni J, Chen YW (2022). Age-dependent oral manifestations of neurofibromatosis type 1: a case-control study. Orphanet J Rare Dis.

[29] Bardellini E, Amadori F, Flocchini P, Conti G, Piana G, Majorana A (2011). Oral findings in 50 children with neurofibromatosis type 1. A case control study. Eur J Paediatr Dent.

[30] Bartzela TN, Carels C, Maltha JC (2017). Update on 13 syndromes affecting craniofacial and dental structures. Front Physiol.

[31] Kobayashi R, Matsune K, Ohashi H (2012). Fused teeth, macrodontia and increased caries are characteristic features of neurofibromatosis type 1 patients with NF1 gene microdeletion. J Pediatr Genet.

[32] Friedrich RE, Giese M, Stelljes C, Froeder C, Scheuer HA (2012). Size of tooth crowns and position of teeth concerning the extension of facial plexiform neurofibroma in patients with neurofibromatosis type 1. Anticancer Res.

[33] Friedrich RE, Grob TJ, Hollants S, Zustin J, Spaepen M, Mautner VF (2016). Recurrent multilocular mandibular giant cell granuloma in neurofibromatosis type 1: Evidence for second hit mutation of NF1 gene in the jaw lesion and treatment with curettage and bone substitute materials. J Craniomaxillofac Surg.

[34] Riecke B, Friedrich RE, Schulze D, Loos C, Blessmann M, Heiland M (2015). Impact of malpositioning on panoramic radiography in implant dentistry. Clin Oral Investig.

[35] Rubenstein LK, Lindauer SJ, Isaacson RJ, Germane N (1991). Development of supernumerary premolars in an orthodontic population. Oral Surg Oral Med Oral Pathol.

